# Mechanisms of YAP/TAZ transcriptional control

**DOI:** 10.15698/cst2021.11.258

**Published:** 2021-10-29

**Authors:** Giusy Battilana, Francesca Zanconato, Stefano Piccolo

**Affiliations:** 1 Department of Molecular Medicine, University of Padua, Via G. Colombo 3, 35131, Padua, Italy.; 2 IFOM, the FIRC Institute of Molecular Oncology, Milan, Italy.

**Keywords:** YAP/TAZ, cancer, Mechanotransduction, Hippo, Brd4, AP-1, proliferation

## Abstract

Dysregulated gene expression is intrinsic to cell transformation, tumorigenesis and metastasis. Cancer-specific gene-expression profiles stem from gene regulatory networks fueled by genetic and epigenetic defects, and by abnormal signals of the tumor microenvironment. These oncogenic signals ultimately engage the transcriptional machinery on the cis -regulatory elements of a host of effector genes, through recruitment of transcription factors (TFs), co-activators and chromatin regulators. That said, whether gene-expression in cancer cells is the chaotic product of myriad regulations or rather a relatively ordered process orchestrated by few TFs (master regulators) has long remained enigmatic. Recent work on the YAP/TAZ co-activators has been instrumental to break new ground into this outstanding issue, revealing that tumor cells hijack growth programs that are active during development and regeneration through engagement of a small set of interconnected TFs and their nuclear partners.

## BASIC CONCEPTS IN YAP/TAZ REGULATION

YAP/TAZ are essential mechanosensors of mammalian cells and are found disproportionally active in most human solid tumors. A wealth of experimental evidence has indicated how YAP/TAZ can drive proliferation and other malignant traits in cancer cells [1]. YAP/TAZ have been classically understood as downstream effectors of the Hippo kinases [2]. However, whether and to what extent the Hippo pathway is in fact regulated in tumors remains unclear and an important area of future investigation. What we do know is that YAP/TAZ activation in tumors is associated to several hallmarks of cancer, such as mutations in RTK/Ras signaling components, altered cell shape and biomechanical changes of the extracellular matrix (ECM; e.g., rigidity, fibrosity etc) [3, 4]. More specifically, cells integrate intracellular oncogenic cues and extracellular mechanical cues to modulate their mechanotransduction [3]. The latter may be defined as the ability of cells to adapt to the physicality of their environment by restructuring the cytoskeleton and the tensional state of the whole cell. While the overarching role of cellular mechanotransduction as ultimate YAP/TAZ regulator is undisputed, the mechanism of this regulation remains unclear, as mechanotransduction seems to incorporate both Hippo-dependent and -independent mechanisms. We refer the reader to more extensive reviews for a more detailed description of the YAP/TAZ upstream control mechanisms [5]. Here, we will focus exclusively on how YAP/TAZ exert their transcriptional effects.

## YAP/TAZ INTERACTION WITH CHROMATIN

### YAP/TAZ transcriptional control through promoters and enhancers

ChIP-seq experiments, thanks to their unbiased nature, have also revealed two unexpected principles of YAP/TAZ transcriptional control. The first one relates to the distribution of YAP/TAZ/TEAD binding sites relative to genes annotated in the human genome. Only a minute fraction of these sites falls on promoters or near to the transcriptional start sites; rather an overwhelming amount (i.e., up to more than 90%) of YAP/TAZ-bound elements correspond to distant enhancer elements [6–11]. The latter also appear in an active state, as revealed by epigenetic marks such as H3K27 acetylation, reduced nucleosome occupancy at the peak center and bimodal distribution of H3K4me1 around the YAP/TAZ peak [6, 9]. Given their “far away” location in the genome, assigning a YAP/TAZ-bound enhancer to its controlled target gene is challenging. Indeed, adopting a proximity criterion (i.e., finding the nearest gene) is questionable, as enhancers can regulate target genes over long distances, physically interacting to their target promoters through chromatin looping, irrespectively of intervening sequences and associated neighbor genes. To overcome this challenge, we have recently integrated YAP/TAZ/TEAD ChIP-seq data with transcriptomic analyses (to identify genes whose expression is addicted to YAP/TAZ activity) and high-resolution (e.g. capture HiC) maps of enhancer-promoter pairs to pinpoint at a comprehensive ensemble of targets and associated cellular processes under direct control of the YAP/TAZ “enhancerome” [6, 12]. A fraction of YAP/TAZ-bound enhancers have the features of superenhancers, with strong enrichment for the binding of transcriptional coactivators and transcription-associated chromatin modifications [10].

### YAP/TAZ transcription and phase separation

It has been proposed that transcriptional coactivators such as MED1 and BRD4, as well as transcription factors, form phase-separated condensates at superenhancers; this results in the concentration of the transcriptional machinery and robust expression of superenhancer-controlled genes [13–15]. Both, overexpressed YAP and TAZ have been shown to form liquid-liquid phase separated bodies on enhancers, which are essential for downstream transcriptional responses [16, 17]. TAZ forms nuclear condensates also containing YAP/TAZ DNA-binding partner TEAD4 (see below), and general transcriptional coactivators MED1, BRD4 and CDK9; the formation of such condensates requires an intact coiled-coil (CC) domain, a function which is not shared by the YAP CC domain [17]. Instead, the domain of YAP which mediates phase separation is the transcriptional activation domain [16]. Recruitment of RNA polymerase II to YAP nuclear condensates seems to be a late event [16], yet, the hierarchy of biochemical events downstream of YAP/TAZ recruitment at cognate enhancers is still poorly understood; for example, it is unknown whether the recruitment of coactivators essential for phase separation, and whether the latter is really causal for transcription or, rather a consequence of YAP/TAZ-catalyzed multiprotein aggregation.

## YAP/TAZ NUCLEAR INTERACTION

### TEAD family members

YAP/TAZ lack an intrinsic DNA-binding domain, and thus can contact the DNA only indirectly through other transcription factors [5]. A number of independent ChIP-seq experiments in different cell lines have established that TEAD family members serve as the dominant DNA-binding platforms for YAP/TAZ. On the genome wide scale, TEAD consensus motifs are indeed found in the vast majority of YAP/TAZ-bound cis-regulatory elements found at promoters and enhancers [6–9, 11, 18, 19].

### Joined transcriptional control by YAP/TAZ and AP-1

A second principle that has emerged from unbiased ChIP-seq data of a variety of cellular contexts is that, after the TEAD consensus, the second most frequent motif at YAP/TAZ bound peaks corresponded to the consensus for AP-1 TFs [6–9, 11, 18, 19]. The latter are dimers of JUN (c-JUN, JUNB, JUND) and FOS (FOS, FOSB, FOSL1 and FOSL2) families of leucine-zipper proteins. In fact, a large fraction of YAP/TAZ/TEAD peaks do also contain an adjoining AP-1 motif, and sequential ChIP-reChIP with first anti-TEAD and then anti-c-JUN antibodies indeed validated that a substantial fraction of the YAP/TAZ cistrome is made by composite TEAD and AP-1 motifs [6]. Biochemically, YAP/TAZ and AP-1 proteins physically interact, either directly (PARK) or indirectly through TEADs [6], suggesting cooperative effects for robust activation of cis-regulatory elements and downstream transcriptional effects. The dual presence of TEAD and AP-1 elements at distal enhancers of YAP/TAZ regulated genes has been also visualized in recent epigenetic studies of human primary colorectal cancer (CRC) cells grown as organoids [11]. The same study also revealed that *de novo* appearance of YAP/TAZ-bound peaks represents an unusually common epigenetic blueprint of all CRCs when compared to normal intestinal tissue, independently of their molecular classification. Strikingly, a core of these YAP/TAZ-fueled deregulated enhancers is also active in diverse tumor types, irrespectively of tissue of origin and genetic aberrations [11].

The above results resonate with those emerging from combined machine-learning and chemicogenomics approaches aimed at the identification of pan-cancer signaling dependencies leading to YAP/TAZ activation [18]. Intriguingly, only dual targeting of YAP and treatment with MEK inhibitors could blunt expression of a conserved set of genes active in different cancer types; the enhancers of these genes were shown to be jointly regulated by YAP/TAZ and AP-1 factors, with MEK inhibitors causing specific loss of AP-1 association to chromatin [18].

The notion that YAP/TAZ and AP1 converge at regulating a core, cancer-specific gene expression program is particularly appealing in light of *in vitro* and *in vivo* results validating this model, and of the many ways by which YAP/TAZ activation feedbacks on expression and regulation of AP1 family members. To start, in *Drosophila*, the YAP/TAZ homologous gene Yorkie contributes to guarantee sufficient expression of ATF3, with loss of Yorkie and AP-1 causing the collapse of the gene-regulatory network sustaining tumor-specific gene signatures initiated by oncogenic Ras [20]. In mammalian cells, AP-1 is crucial for YAP/TAZ driven transformation and induction of tumorigenic potential of mammary cells [6]. Conversely, YAP/TAZ are genetically required for tumor emergence after application of the skin chemical carcinogenesis protocol, consisting of tumor initiation, leading to oncogenic Ras followed by tumor promotion with phorbol ester known to operate through AP-1 activation [6]. More directly, oncogenic activation of YAP/TAZ after genetic loss of Hippo kinases LATS1/2 causes robust induction of AP-1 target genes, with pharmacological inhibition of AP-1 causing attenuation of YAP/TAZ-driven transformation of pancreatic cells *in vivo* [19].

AP-1 is not only a transcriptional partner of YAP/TAZ but also, in a typical self-sustaining positive feedback loop, a transcriptional target of YAP/TAZ. This loop has been documented in several contexts. For example, YAP/TAZ directly promote FOS transcription, in turn contributing to the YAP/TAZ-controlled gene expression program [21, 22].

Inhibition of AP-1 blunts YAP/TAZ-driven tumorigenesis induced by inactivation of the Hippo kinases LAST1/2, and liver overgrowth *in vivo* caused by overexpression of YAP itself [21]. Moreover, in Basal Cell Carcinoma, YAP potentiates c-JUN mRNA and protein stability by sustaining c-JUN phosphorylation by JNK [23]. These results start to paint a picture in which YAP/TAZ activation downstream of oncogenic insults (in particular RTK/Ras signaling [3]) and altered cell mechanics controls cancer-specific gene expression in concert with AP-1, by direct transcriptional cooperation at cis-regulatory elements and by controlling expression of AP-1 family members. What remains unclear is whether the converse may be also true, that is, whether YAP/TAZ mRNA expression may be fueled by AP-1. Unfortunately, little is known on the transcriptional control of YAP and TAZ, but recent work in gastric cancer cell lines indeed hinted to the possibility that MAPK signaling sustains expression of YAP mRNA through c-JUN [24], yet through unclear mechanisms.

The widespread presence of AP-1 sites at YAP/TAZ regulated genes raises questions on to what extent targeting the YAP/TAZ-TEAD interaction may be in fact sufficient to blunt YAP/TAZ-driven tumorigenesis. YAP has been indeed shown to bind directly to AP-1, and recent work by K. Struhl and colleagues proposes that YAP/TAZ may serve as transcriptional co-activators of JUNB to control a set of genes active during cell transformation, but that only in part overlap with those regulated by YAP/TAZ/TEAD [25]. Clearly, more work is required to dissect the cooperative vs. potentially redundant roles of TEAD and AP-1, and to gauge the contribution of AP-1 factors to YAP/TAZ biology in cancer.

### YAP/TAZ interaction with general transcriptional coactivators

Binding of YAP/TAZ to chromatin is just the first of a series of biochemical steps eventually culminating in RNA polymerase II (Pol2) recruitment and activation of YAP/TAZ-driven transcription. To start, YAP/TAZ recruit the general coactivator bromodomain-containing protein 4 (BRD4), in fact dictating the genome-wide association of BRD4 to chromatin, and endowing to YAP/TAZ-bound enhancers the same functional attributes of superenhancers [7]. Thus, YAP/TAZ-bound enhancers mediate *de novo* recruitment of Pol2 at YAP/TAZ-regulated promoters. Moreover, YAP/TAZ also contact Pol2 through the Mediator complex (MED1), promoting CDK9-mediated phosphorylation of the Pol2 C-terminal tail, as such favoring transcriptional pause-release [10]. All in all, these epigenetic steps are essential for activation of cell proliferation.

**Figure 1:**
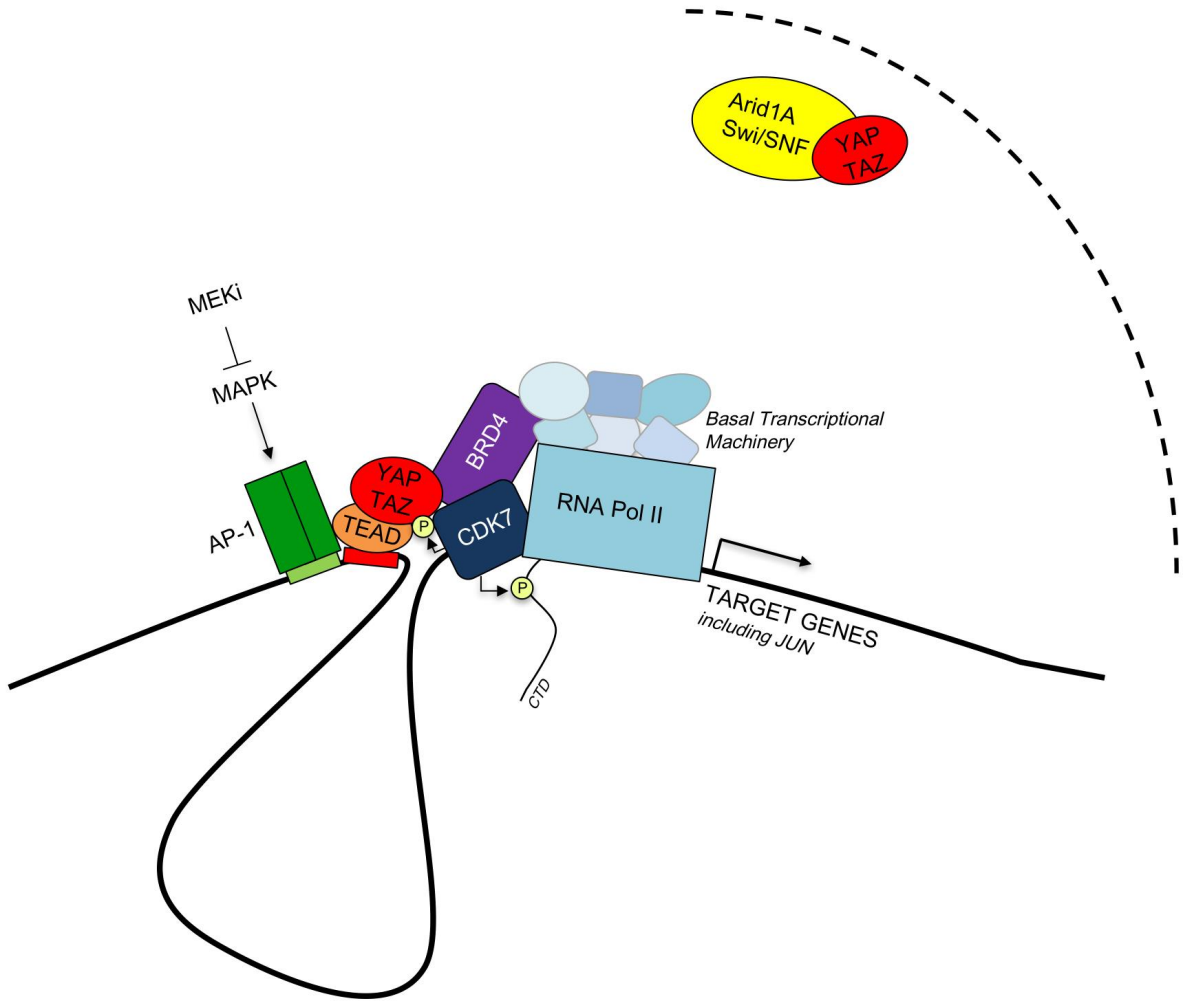
FIGURE 1: Model of the interactions between YAP/TAZ, partner transcription factors, epigenetic modulators and the basal transcriptional machinery (See text for details).

YAP/TAZ has been recently shown to interact also with CDK7, a component of the basal transcriptional machinery [26]. Interestingly, CDK7 directly phosphorylates YAP/TAZ in a Hippo-independent manner, preventing their degradation by nuclear ubiquitin ligases. This step promotes YAP/TAZ association to enhancer elements, raising prospects to use CDK7 inhibitors as YAP/TAZ inhibitory drugs. The YAP/TAZ – CDK7 connection also explains why CDK7 inhibitors disproportionally inhibit the expression of genes controlled by superenhancers [26], that would be instead at odd with the traditional function of CDK7 as element of the basal transcriptional machinery. A potentially unifying model, yet so far speculative, may be one in which CDK7, by stabilizing YAP/TAZ at their bound chromatin sites, would favor robust recruitment at the same sites of BRD4, leading to YAP/TAZ transcriptional addictions in tumor cells. Irrespectively, these findings offer fresh insights on how to target YAP/TAZ in cancer and a host of potential new applications for already existing drugs, such as BET-inhibitors (targeting Brd4 and related factors) or CDK7. For example, treatment with small-molecule inhibitors of BRD4 blunts YAP/TAZ pro-tumorigenic activity in several experimental models [7].

### Buffering YAP/TAZ activity by YAP/TAZ-ARID1A/SWI/SNF

Finally, YAP/TAZ nuclear functions are buffered by an inhibitory association with the BAF-SWI/SNF complex, through the ARID1A subunit [27]. Of note, this interaction is controlled by mechanotransduction, in the sense that the ARID1a-YAP/TAZ association is favored by low mechanical strains (that correspond to the more physiological/homeostatic tissue conditions) and disrupted when cells are exposed to elevated mechanical strains, as it occurs in cancer or during regeneration, that is, when cells are exposed to an abnormally rigid ECM on which to spread. Indeed, under these conditions ARID1A binds to F-actin, unleashing YAP/TAZ activity [27]. The findings indicate that loss of ARID1a, that is frequent in tumor, may increase mechanotransduction. In other words, full activation of YAP/TAZ requires not only nuclear accumulation of YAP/TAZ but also, as permissive step, overcoming the ARID1A-SWI/SNF barrier; the latter may occur by genetic-deletion of ARID1a or by the intrinsically altered physicality of the tumor microenvironment. The results are consistent with the role of ARID1a as tumor suppressor and with genetic evidence indicating that attenuation of ARID1a levels potently fosters cell regeneration with *in vivo* phenotypes recapitulating those ascribed to YAP/TAZ activation, such as pancreatic acinar to ductal metaplasia, liver regeneration and cardiomyocyte proliferation [28]. That said, a direct epistatic connection between loss of ARID1a and YAP/TAZ during tissue regeneration remains to be demonstrated. Also undefined is the chromatin context in which BAF-SWI/SNF regulates YAP/TAZ. In this respect, it is worth mentioning that AP-1 has been shown to serve as pioneer factor in fact by recruiting SWI/SNF at enhancer elements [29]. This raises the tempting possibility that AP1 may on the one hand open up the YAP/TAZ cistrome, and, on the other, fine-tunes transcription from those enhancers through the locally recruited SWI/SNF, buffering against sub-threshold fluctuations of nuclear YAP/TAZ levels, as such ensuring that robust YAP/TAZ target gene transcription would occur only under appropriate conditions.

## THERAPEUTIC STRATEGIES FROM THE MECHANISMS OF YAP/TAZ TRANSCRIPTIONAL CONTROL

The ability of YAP/TAZ to sustain tumorigenesis *in vivo* and in a variety of organs and tissue types, coupled with the apparent dispensability of YAP/TAZ for the normal homeostasis of those same organs, has fueled translational research aimed to develop drugs specifically targeting YAP/TAZ function [30]. The disordered nature of YAP/TAZ proteins complicates their targeting by small molecules. However, YAP/TAZ reliance on other proteins at cognate cis-regulatory elements has opened several opportunities to indirectly interfere with their activity. One particularly appealing route of intervention is small molecules targeting TEAD palmitoylation, in principle leading to pan-TEAD instability and loss of YAP/TAZ/TEAD association to chromatin [31]. As mentioned above, inhibitors of CDK9, CDK7 or BET proteins along with other epigenetic modulators (HDACs) may represent additional routes to interfere with YAP/TAZ function, an approach that is obviously less specific if compared to TEAD targeting, but based on already existing and clinically validated compounds [30]. Then, the pervasive roles of AP-1 in YAP/TAZ-driven transcription also offers the possibility to repurpose as indirect YAP/TAZ inhibitors a number of drugs known to impinge on AP-1 activity or on JUN/FOS expression and stability. In this perspective, it would be interesting to investigate to what extent inhibitors of the Ras cascade and of MAPK might be repurposed as indirect YAP/TAZ inhibitors, including RasG12V-, Raf-, JNK-, and MEK-inhibitors. Evidence in this direction is accumulating [30], although it remains unclear to what extent these YAP/TAZ attenuating effects are mediated by AP-1 inhibition and/or other responses (including a role for Ras/MAPK signaling on the cytoskeleton [3]).

In conclusion, research on YAP/TAZ transcriptional mechanisms has started to shed some light on essential mediators, mechanisms and genome-wide regulatory elements that are critical for YAP/TAZ biology, laying the groundwork for new routes of pharmacological interventions aimed at controlling YAP/TAZ responses *in vivo*.

## Abbreviatons:

CC: – coiled-coil,
CRC: – colorectal cancer,
ECM: – extracellular matrix,
Pol2: – RNA polymerase II.
